# An Unexpected Interaction between Sofosbuvir/Ledipasvir and Atorvastatin and Colchicine Causing Rhabdomyolysis in a Patient with Impaired Renal Function

**DOI:** 10.1155/2016/3191089

**Published:** 2016-08-22

**Authors:** Shyam Patel, Jennifer Andres, Kamran Qureshi

**Affiliations:** ^1^Department of Medicine, Temple University Hospital, Philadelphia, PA 19140, USA; ^2^Department of Pharmacy Practice, Temple University School of Pharmacy, Philadelphia, PA 19140, USA; ^3^Department of Medicine, Section of Gastroenterology, Temple University Lewis Katz School of Medicine, 3440 N. Broad Street, Philadelphia, PA 19140, USA

## Abstract

Hepatitis C virus (HCV) infection affects roughly 170 million people worldwide. Sofosbuvir/Ledipasvir (Sof/Led) is a new once daily direct acting antiviral combination pill that was approved in October 2014 for use in patients with HCV genotype 1 infection. Coadministration of Sof/Led is studied only with rosuvastatin which shows significantly increased level of drug and is associated with increased risk of myopathy, including rhabdomyolysis. There is no mention of such HMG-CoA reductase inhibitor interaction as a class, as pravastatin did not have any clinically significant interaction with Sof/Led. Other myotoxic drugs, including colchicine are not studied. We present a case of a serious drug interaction between Sof/Led and atorvastatin, in the background of CKD and colchicine use.

## 1. Case Report

We present a case of 66-year-old African American female with medical history of compensated liver cirrhosis related to chronic hepatitis C virus (HCV) genotype 1a infection, stage III Chronic Kidney Disease (CKD) related to hypertensive nephropathy, hypertension, coronary artery disease, and gout, who was recommended to start Sofosbuvir/Ledipasvir (Sof/Led) as 400/90 mg tablet along with renal dosed ribavirin 200 mg twice daily for 12 weeks for her HCV infection. The regimen was selected based on the presence of biopsy proven early cirrhosis and HCV infection, which was treated in the past with pegylated interferon, telaprevir, and ribavirin (she relapsed after early termination of this regimen because of intolerable side effects). Two weeks prior to the initiation of current treatment attempt with Sof/Led and ribavirin, baseline labs were unremarkable, as indicated in Table 1. Her daily home medications colchicine 0.6 mg, atorvastatin 80 mg, allopurinol, clonidine, lisinopril, labetalol, and aspirin were continued. It is unclear how long the patient had been on the colchicine and atorvastatin, but the patient had mentioned that these two medications had been chronic medications that she had been on for over 5 years.

**Figure 1:**
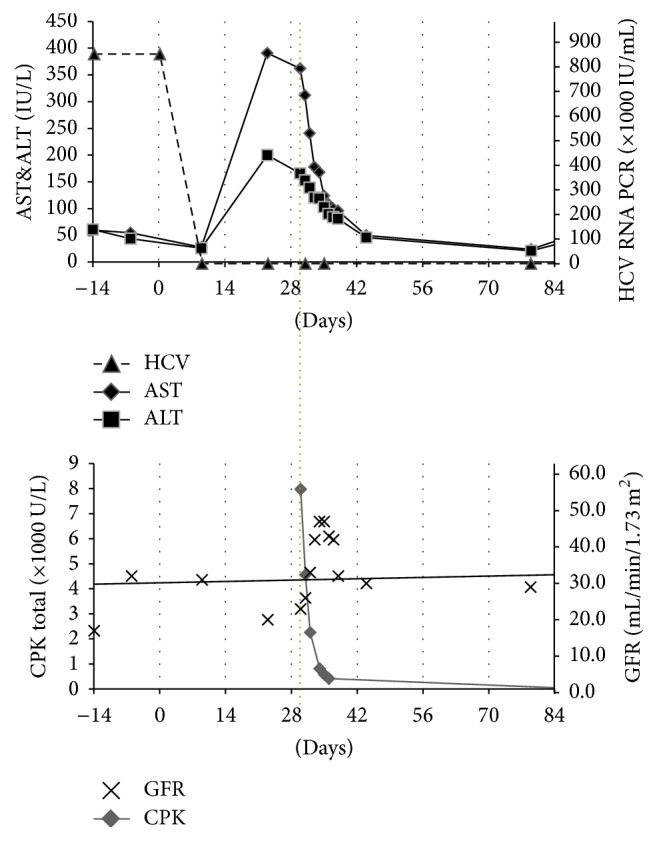
Timeline of laboratory changes. Interrupted line denotes hospital admission day.

Three weeks after starting therapy, she noticed nausea, vomiting, diarrhea, and nonspecific abdominal pain. Blood tests at that time (as indicated in Figure 1, day 23), showed undetectable HCV viral load, with new elevations of liver enzymes and she was advised to increase oral hydration for symptomatic relief. One week later (day 30), she presented to emergency room with complaints of profound weakness and myalgia along with ongoing nausea and abdominal pain. On physical examination, she appeared slightly dry, with slight periumbilical tenderness and without ascites. She demonstrated diffuse muscle weakness and muscle tenderness in all extremities. No focal neurologic deficit was identified. The admission labs (as noted in Table 1 and Figure 1, day 30) were noticeable for elevated liver enzymes, creatinine, and creatine phosphokinase (CPK). Urinalysis showed large amount of blood, without RBCs. Clinical diagnosis of rhabdomyolysis was made. Her atorvastatin was stopped and she was aggressively fluid resuscitated. CPK levels subsequently trended down and renal function returned to baseline. The workup for myalgia was unremarkable, with normal aldolase, C3 and C4 complement levels, TSH, and serum protein electrophoresis. Neurology evaluation was done, and EMG was done to evaluate for profound muscular weakness. EMG showed diffuse muscle irritability with no evidence of polyneuropathy, suggesting this patient had an inflammatory myopathy.

Rhabdomyolysis was considered likely from an interaction between Sof/Led, atorvastatin, and colchicine in the background of abnormal renal function. The rise of AST/ALT was considered to be from her rhabdomyolysis (nonhepatic). Colchicine was also stopped and she was continued on Sof/Led and low dose ribavirin with daily monitoring of her labs. By the time of discharge, her rhabdomyolysis resolved, with return of baseline GFR of 43 mL/min/1.73 m^2^. Although her myalgia improved, she was discharged home with persistent muscle weakness, which subsequently improved over the next 4 weeks with physical rehabilitation. She successfully completed 12-week course of HCV therapy and did not have any recurrent symptoms or findings of rhabdomyolysis as she was kept off atorvastatin and colchicine. Her muscle weakness had resolved by the time she finished her therapy. Her follow-up labs showed SVR12 (Table 1); thus, her HCV infection was considered cured and her renal function and other laboratory testing returned to baseline.

## 2. Discussion

On October 10, 2014, the FDA approved the fixed-dose combination pill Sof/Led (Harvoni, Gilead Sciences) for the treatment of chronic HCV genotype 1 infection [1]. Sof is a nucleotide NS5B polymerase inhibitor and Led is an NS5A inhibitor for HCV [2]. The common side effects of Sof/Led are fatigue and headache. Interestingly, benign, asymptomatic elevation of CPK has been reported in patients taking Sof/ribavirin or interferon/ribavirin [3].

There have been no case reports of Sof/Led interactions with atorvastatin and colchicine in causing rhabdomyolysis. In addition, the Sof/Led label does not mention many drug interactions; however both components are P-gp substrates [4]. Led is an inhibitor of the drug transporters P-gp and Breast Cancer Resistance Protein (BCRP) and may increase intestinal absorption of coadministered substrates for these transporters [5]. Sof/Led is not recommended to be given with other known P-gp-inducers due to the potential for decrease in efficacy. Led can increase absorption of colchicine by P-gp inhibition and of rosuvastatin via inhibition of BCRP. Coadministration of Sof/Led with rosuvastatin can potentially increase the risk of statin related myopathy and rhabdomyolysis [6]. Sof accumulation in renal failure can also lead to toxicity and the patient should be closely monitored for liver failure if Sof is to be used in these patients. Additionally, colchicine has the independent ability to cause myotoxicity [7]. Sof/Led has not been noted to interact adversely with atorvastatin; however, a theoretical interaction exists via P-gp inhibition by atorvastatin, causing an increased level of led. Atorvastatin can itself increase levels of colchicine due to P-gp inhibition. Using colchicine in combination with a statin has been reported to cause myotoxicity, especially in the setting of CKD [8]. Atorvastatin 80 mg was used in this patient as it was used for secondary prevention for her coronary artery disease. A lower dose of atorvastatin was not attempted as the patient was symptomatic from her rhabdomyolysis and the patient was not having any active coronary artery disease.

In our patient, the addition of the Led in the presence of atorvastatin could have further increased the levels of colchicine, increasing the likelihood of muscle toxicity and causing rhabdomyolysis. Colchicine is renally eliminated and requires dose adjustments based on renal dysfunction. Thus, this patient's stage III CKD combined with a high colchicine dose and P-gp mediated drug interactions could have played a critical role in increasing the chance of this unanticipated toxicity [8]. There was little concern for mitochondrial toxicity caused by a lactic acidosis as the patient has a normal anion gap and normal lactate levels.

The Naranjo probability score [9] was developed to help assess causality for adverse drug reactions and based on score of 6 (Table 2) this patient's adverse event correlates with a probable adverse drug reaction. Although atorvastatin levels were directly measured, the muscle toxicity described was likely attributed to drug interactions in combination with preexisting renal impairment. Sof/Led was coadministered with two drugs known to cause myositis. Drug interactions, via P-gp inhibition, caused increased levels of all drugs, potentially increasing the risk of muscle problem development. It is important for physicians' prescribing Sof/Led to be aware of the potential drug interactions.

Appropriate pharmacy evaluation and medication changes should be made in such cases to avoid untoward reactions from drug interactions, including myositis and rhabdomyolysis as in our case. If rhabdomyolysis is discovered in a patient taking atorvastatin, Sof/Led, and/or colchicine, it should be treated with IV fluids and the CK levels should be trended. In addition, colchicine should be stopped, as there are other treatments for an acute gout flare (such as prednisone) that do not have a drug-drug interaction with atorvastatin and Sof/Led in causing rhabdomyolysis. In the setting of symptomatic rhabdomyolysis in a patient with no active coronary disease, atorvastatin should be stopped and can be resumed once the Sof/Led therapy is completed.

## Figures and Tables

**Table 1:** Pertinent laboratory results.

	Baseline	Admission	SVR12
WBC (×1000/*μ*L)	5.3	2.2	3.6
Hgb (gm/dL)	11.5	10	11.2
Plt (×1000/*μ*L)	140	58	142
Tbili (mg/dL)	0.4	1.6	0.5
ALT (U/L)	61	166	20
AST (U/L)	60	362	24
Cr	1.87	2.5	1.88
GFR (mL/min/1.73 m^2^)	32	23	32
UA		Large blood, 0 RBC	Negative blood
CPK (U/L)		7979	65
HCV RNA PCR (IU/mL)	8517000	Undetectable	Undetectable

**Table 2:** Naranjo probability scale. Total scores range from −4 to +13; the reaction is considered definite if the score is 9 or higher, probable if 5 to 8, possible if 1 to 4, and doubtful if 0 or less.

	Yes	No	Do not know
Are there previous conclusive reports of this reaction?	+1	0	0

Did the adverse event appear after the drug was given?	+2	−1	0

Did the adverse reaction improve when the drug was discontinued or a specific antagonist was given?	+1	0	0

Did the adverse reaction reappear upon readministering the drug?	+2	−2	0

Were there other possible causes for the reaction?	−1	+2	0

Did the adverse reaction reappear upon administration of placebo?	−1	+1	0

Was the drug detected in the blood or were other fluids detected in toxic concentrations?	+1	0	0

Was the reaction worsened upon increasing the dose? Or was the reaction lessened upon decreasing the dose?	+1	0	0

Did the patient have a similar reaction to the drug or a related agent in the past?	+1	0	0

Was the adverse event confirmed by any other objective evidence?	+1	0	0
